# Associations between behavioral trait expression, anxiety, and depression in a multi-site recovery sample

**DOI:** 10.3389/fpsyg.2026.1871291

**Published:** 2026-10-06

**Authors:** Jason Roop

**Affiliations:** Center for Trait-Based Transformation, Louisville, KY, United States

**Keywords:** anxiety, behavioral health, depression, observational study, participant-level analysis, psychological distress, temporal dynamics, trait expression

## Abstract

**Background:**

Anxiety and depressive symptoms are commonly evaluated as indicators of psychological distress, yet less is known about how broader patterns of behavioral trait expression relate to these symptoms at the individual level. This study examined associations between overall trait expression and anxiety and depression among adults participating in recovery-oriented behavioral health programming.

**Methods:**

A retrospective observational analysis was conducted using routine assessment data collected between August 2023 and May 2026 across multiple U. S. behavioral healthcare settings. Records from the Trait and Hero Discovery Assessment, Generalized Anxiety Disorder scale (GAD-7), and Patient Health Questionnaire (PHQ-9) were matched to the same participant and assessment occasion. The primary analytic sample included 278 matched assessment occasions representing 252 participants. Associations were evaluated using Pearson correlations, with Spearman correlations, participant-clustered regression, mixed-effects models, and alternative assessment-matching windows used as sensitivity analyses. Internal consistency was also evaluated for the study measures.

**Results:**

Higher Trait Index scores were moderately associated with lower anxiety (*r* = −0.471, 95% CI [−0.557, −0.372], *p* < 0.001) and lower depressive symptom severity (*r* = −0.508, 95% CI [−0.595, −0.408], *p* < 0.001). Findings remained consistent across nonparametric, clustered, mixed-effects, and alternative matching-window analyses. The strength of the trait-depression association did not differ reliably from the trait-anxiety association. Internal consistency was strong for the overall Trait Index (Cronbach’s alpha = 0.901), GAD-7 (alpha = 0.922), and PHQ-9 (alpha = 0.925). Exploratory analyses of participants with repeated matched assessments did not establish temporal precedence between trait expression and psychological distress.

**Conclusion:**

Greater expression of the assessed behavioral traits was consistently associated with lower anxiety and depressive symptoms at the individual level. These observational findings do not establish causality or directionality, but they support further longitudinal investigation into whether trait expression functions as an antecedent, consequence, or reciprocal correlate of psychological distress. The findings also suggest potential value in complementing symptom-focused assessment with attention to underlying behavioral strengths and capacities in applied behavioral health settings.

## Introduction

Anxiety and depressive symptoms are common dimensions of psychological distress and contribute substantially to global mental-health burden (GBD 2019 Mental Disorders Collaborators, 2022). Clinical assessment appropriately emphasizes symptom severity, functional impairment, and diagnostic formulation. At the same time, symptom scores do not fully explain why individuals exposed to similar stressors differ in emotional response, adaptation, or recovery. Contemporary psychological frameworks increasingly conceptualize mental health as emerging from interacting processes rather than from isolated symptoms alone (Borsboom, 2017; Hayes et al., 2020). This has renewed interest in measurable individual characteristics that may shape how people respond to stress and psychological challenge.

Personality and trait research provides one relevant foundation (McCrae and Costa, 2008). Traits are comparatively stable patterns of thinking, feeling, and behavior, but stability does not imply immutability. Longitudinal and intervention research indicates that trait expression can vary with development, context, psychiatric state, and intentional change (Roberts et al., 2006; Prince et al., 2021; Stieger et al., 2021). Recent prospective work further suggests that personality characteristics can contribute information about later mental health and functioning beyond prior diagnostic history, while psychiatric symptoms may themselves alter trait expression over time (Prince et al., 2021; Waszczuk et al., 2022). These findings are consistent with vulnerability, complication/scar, and reciprocal models rather than a simple one-directional account.

The relationship between traits and psychological distress is also unlikely to be uniform across symptom dimensions. Kang et al. (2023), using a large population sample, found that Big Five traits were differentially associated with distinct dimensions of mental health. Conway et al. (2024) similarly demonstrated that neuroticism/negative emotionality was prospectively associated with change in broad internalizing symptoms, while relationships with narrower symptom dimensions differed. Such work supports examining both broad trait configurations and specific forms of distress rather than assuming that anxiety and depression reflect identical underlying processes.

The present study evaluates a different, applied trait framework developed for recovery-oriented behavioral health programming. The Trait and Hero Discovery Assessment organizes functioning across 10 behavioral domains: tenacity, resilience, empathy, authenticity, motivational orientation, creativity, appreciation, self-awareness, determination, and emotional intelligence. These domains are not intended as substitutes for established personality taxonomies or clinical symptom measures. Rather, they operationalize a strengths-oriented set of behavioral capacities used within Trait-Based programming. An overall Trait Index summarizes expression across the 10 domains, while the GAD-7 and PHQ-9 independently assess anxiety and depressive symptom severity.

Assessments were administered at developmental or program milestones rather than on a fixed monthly schedule. Calendar month therefore did not represent a repeated-measures time unit. The analysis linked records to the same participant and assessment occasion and accounted explicitly for repeated observations.

The revised study addresses three primary objectives: (1) to determine whether the 10-domain Trait Index is associated with GAD-7 and PHQ-9 symptom severity at matched participant assessment occasions; (2) to evaluate the robustness of these associations across nonparametric, clustered, mixed-effects, adjusted, and alternative matching-window analyses; and (3) to explore, among participants with repeated complete assessment occasions, whether the available data provide preliminary evidence regarding temporal direction. Because the design is observational, all directional analyses are considered exploratory and no causal sequence is assumed.

## Materials and methods

### Study design and data source

This study used a retrospective observational design based on assessment records collected during routine Trait-Based programming in recovery-oriented behavioral healthcare settings. Source records spanned August 2023 through May 2026 and originated from participating U. S. sites across seven states. The assessments were administered as part of ordinary program implementation within behavioral healthcare treatment settings with provider authorization; no new recruitment, intervention, or participant contact was undertaken for the present secondary analysis.

The original exports contained 2,331 Recovery Trait Assessment administrations, 706 GAD-7 administrations, and 611 PHQ-9 administrations. Records were audited at the item and score levels before linkage. Raw source exports were preserved unchanged, and all cleaning, score reconstruction, matching, and analysis were performed in derivative working files.

### Ethics and privacy

Campbellsville University reviewed the revised secondary analysis and, on August 16, 2026, determined that it qualified as exempt secondary research under 45 CFR 46.104(d)(4)(iii). No protocol or determination number was assigned. The determination confirmed that no new informed-consent process was required for this secondary analysis under the selected exemption. The study was conducted in accordance with applicable institutional and federal ethical requirements and the ethical principles of the Declaration of Helsinki as applicable to retrospective secondary research. Direct identifiers were used only temporarily for record linkage in a secure working environment; the analytic dataset used coded study identifiers, and results are reported only in aggregate. No participant was contacted or re-identified for the research analysis. Applicable obligations under HIPAA, 42 CFR Part 2, provider agreements, institutional policy, and state law were preserved.

### Measures

#### Trait and Hero discovery assessment

Trait expression was assessed with the Trait and Hero Discovery Assessment, a multidimensional self-report instrument developed for Trait-Based programming. The current administration form contains 53 scored items distributed across 10 behavioral domains: tenacity, resilience, empathy, authenticity, motivational orientation, creativity, appreciation, self-awareness, determination, and emotional intelligence. Items use Likert-type response formats, including positively and negatively worded items, and are scored within the assessment system to yield standardized domain scores on a common 0–100 metric, with higher scores indicating greater expression of the assessed capacity. Minor wording and item-set adaptations occurred during implementation; therefore, item-level domain reliability analyses were restricted to the identifiable current-form administrations, whereas the standardized 10-domain composite could be computed across the full valid Recovery dataset.

For the present study, the Trait Index was recomputed as the arithmetic mean of the 10 standardized domain scores. The platform-exported Average Score was not used because it also included Hero/archetype scores and therefore did not correspond exactly to the 10-domain construct described in the manuscript. Hero/archetype scores were not analyzed. Across all 2,329 valid Recovery Trait administrations, the 10-domain Trait Index demonstrated strong internal consistency (Cronbach’s alpha = 0.901, bootstrap 95% CI [0.893, 0.907]; omega total = 0.901). In the identifiable current form, composite alpha and omega were both 0.918. Domain-level reliability estimates are reported in the Results and interpreted cautiously for lower-reliability scales.

#### Generalized anxiety disorder scale

Anxiety symptom severity was measured with the Generalized Anxiety Disorder scale (GAD-7; Spitzer et al., 2006). Seven items are rated from 0 (not at all) to 3 (nearly every day), producing a total score from 0 to 21. Higher scores indicate greater anxiety symptom severity. Current-sample internal consistency was strong (Cronbach’s alpha = 0.922, bootstrap 95% CI [0.912, 0.931]).

#### Patient health questionnaire

Depressive symptom severity was measured with the Patient Health Questionnaire (PHQ-9; Kroenke et al., 2001). Nine items are rated from 0 (not at all) to 3 (nearly every day), producing a total score from 0 to 27. Higher scores indicate greater depressive symptom severity. Current-sample internal consistency was strong (Cronbach’s alpha = 0.925, bootstrap 95% CI [0.914, 0.934]).

### Data processing, score reconstruction, and matching

Two Trait Assessment records contained impossible domain/composite values and were excluded, leaving 2,329 valid Trait administrations. GAD-7 and PHQ-9 totals were reconstructed from item responses because the platform export labeled some all-zero administrations as “Incomplete.” Seventy-four GAD-7 and 67 PHQ-9 administrations consisted of valid zero responses across all symptom items and were retained. All 706 GAD-7 records contained valid 0–3 item responses. Twenty PHQ-9 records contained impossible item values above 3 caused by multiple selections on one or more items and were excluded under a prespecified validity rule, leaving 591 valid PHQ-9 administrations. No observation was excluded merely because a valid score was unusually high or low.

Records were then linked across the three Recovery instruments to create same-participant, same-occasion triads. The primary matching rule required the same normalized participant identity across instruments, corroborating age and email information, a maximum of 60 min between the earliest and latest assessment completion time, and one-to-one matching so that no administration was reused. Every retained primary triad had matching age and at least two matching email records; 268 of 278 had the same email across all three instruments. When two complete triads occurred within 24 h for the same participant, the earliest triad was retained and the immediate retest was excluded. Matching windows of 30 min, 2 h, 6 h, 12 h, and 24 h were evaluated as sensitivity analyses.

The matching process identified 280 initial triads. Two immediate same-day retest triads were excluded, yielding 278 matched assessment occasions from 252 participants. Of these participants, 229 contributed one matched occasion, 20 contributed two, and 3 contributed three.

### Statistical analysis

Descriptive statistics were calculated for the full valid Trait, GAD-7, and PHQ-9 datasets, including mean, median, standard deviation, range, skewness, and excess kurtosis. Primary associations between Trait Index and each symptom measure were estimated using Pearson correlations at the matched assessment-occasion level. Two-sided *p*-values and participant-cluster bootstrap 95% confidence intervals were reported to account for the small number of participants contributing repeated occasions. Spearman rank correlations were used as nonparametric sensitivity analyses.

Cluster-robust linear regressions quantified symptom-score differences per 10-point higher Trait Index. Additional sensitivity models adjusted for site and reported program module/stage where available, and random-intercept mixed-effects models were fitted with participant as the grouping variable. A participant-cluster bootstrap was used to compare the magnitudes of the Trait-GAD-7 and Trait-PHQ-9 correlations directly. Quadratic terms were tested to evaluate whether a linear association was an adequate first approximation over the observed Trait Index range. Alternative matching-window analyses assessed the stability of the primary result.

Internal consistency was evaluated using Cronbach’s alpha with bootstrap confidence intervals. Omega total and principal-component structure were also examined for the higher-order 10-domain Trait Index. Domain-level reliability was estimated for the identifiable current Trait Assessment form. Repeated complete matched occasions were analyzed separately and explicitly labeled exploratory. First-to-last paired changes were summarized, and exploratory lagged regressions assessed whether earlier Trait Index predicted later GAD-7 or PHQ-9 after controlling for the prior symptom value and interval, and whether the reverse paths were present. The repeated subset was considered too small for definitive temporal inference.

Analyses were conducted in Python 3.13.5 using pandas 2.2.3, NumPy 2.3.5, SciPy 1.17.0, statsmodels 0.14.6, and scikit-learn 1.8.0. Statistical tests were two-sided with alpha = 0.05. Effect sizes and confidence intervals were emphasized in interpretation.

## Results

### Data flow and descriptive characteristics

The Recovery Trait dataset contained 2,331 raw administrations, of which 2,329 met validity criteria. All 706 GAD-7 administrations were retained after item-level score reconstruction. Of 611 PHQ-9 administrations, 591 were valid after excluding 20 records with impossible multi-selection item values. The primary linkage process yielded 278 matched Trait-GAD-7-PHQ-9 assessment occasions representing 252 participants (Figure 1; Table 1).

**Figure 1:**
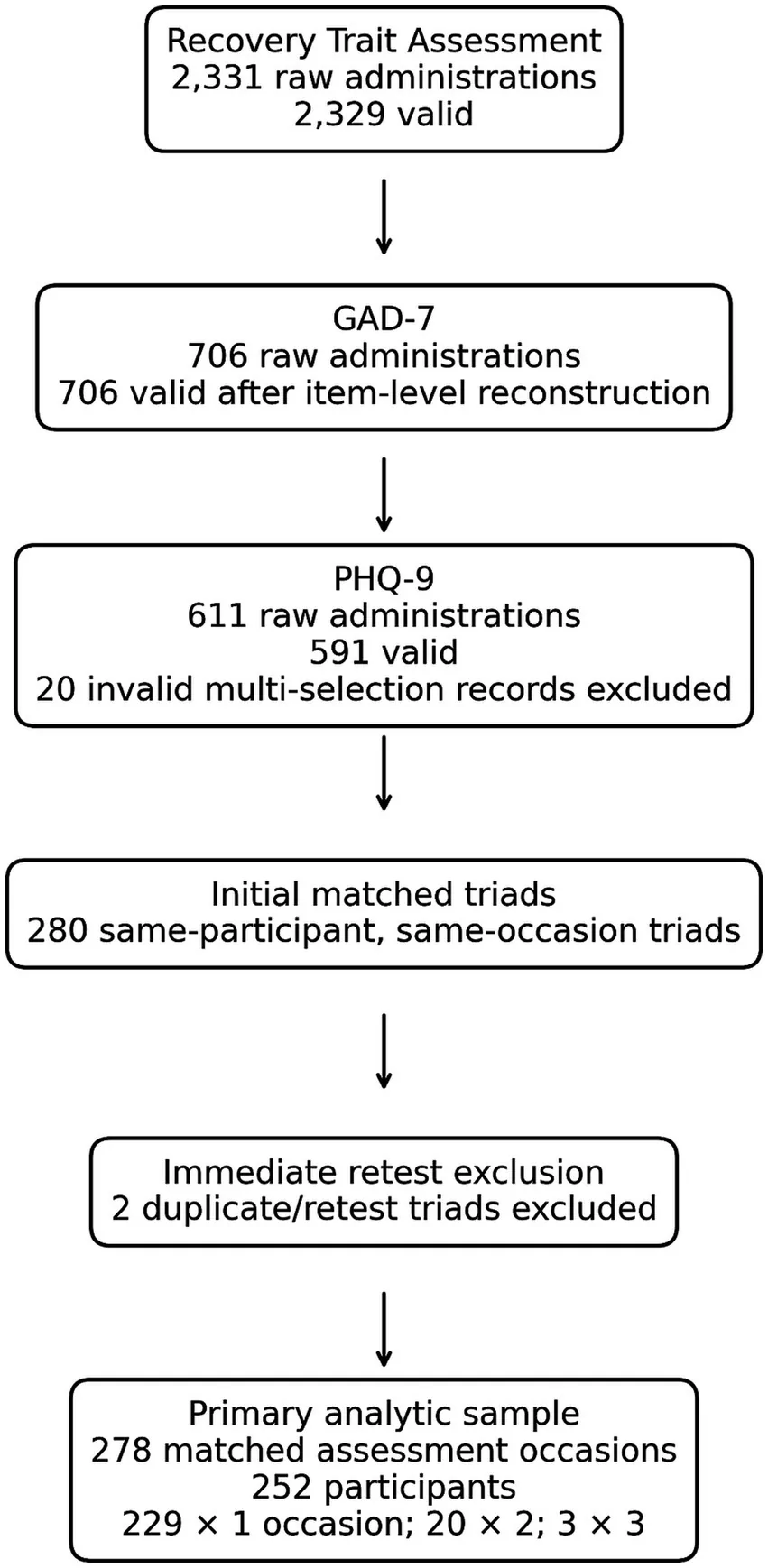
Data flow for the revised participant-level analysis. Symptom totals were reconstructed from valid item-level responses before participant-level matching. The primary sample contained 278 matched assessment occasions from 252 participants.

**Table 1:** Data audit and primary analytic flow.

Dataset / stage	Raw *N*	Valid / Retained *N*	Primary reason for exclusion or correction
Recovery Trait Assessment	2,331	2,329	2 records with impossible trait-domain/composite values
GAD-7	706	706	Valid zero totals reconstructed from item responses and retained
PHQ-9	611	591	20 records with impossible multi-selection item values excluded
Initial matched triads	280	280	Same participant; <=60-min assessment span
Immediate retests	2	0	Later duplicate/retest triad within 24 h excluded
Primary analytic sample	-	278 occasions / 252 participants	229 participants x1; 20 ×2; 3 ×3

Across all valid Recovery Trait administrations, the Trait Index had a mean of 69.35 (SD = 13.34), median of 70.10, and range of 8 to 98. The distribution was moderately negatively skewed (skewness = −0.68) with modest positive excess kurtosis (1.11). Across all valid GAD-7 administrations, the mean was 8.14 (SD = 6.18; median = 7; range = 0–21). Across valid PHQ-9 administrations, the mean was 9.26 (SD = 7.79; median = 7; range = 0–27). Full descriptive statistics are shown in Table 2.

**Table 2:** Descriptive statistics for valid source assessments.

Measure	*N*	Mean	Median	SD	Range	Skew	Excess kurtosis	Alpha
Trait Index	2,329	69.35	70.10	13.34	8–98	−0.68	1.11	0.901
GAD-7	706	8.14	7.00	6.18	0–21	0.49	−0.85	0.922
PHQ-9	591	9.26	7.00	7.79	0–27	0.68	−0.62	0.925

### Primary associations with psychological distress

At matched assessment occasions, higher Trait Index scores were moderately associated with lower anxiety symptom severity (*r* = −0.471, participant-cluster bootstrap 95% CI [−0.557, −0.372], *p* < 0.001) and lower depressive symptom severity (*r* = −0.508, 95% CI [−0.595, −0.408], *p* < 0.001). Spearman correlations were nearly identical (rho = −0.469 for GAD-7; rho = −0.519 for PHQ-9), indicating that the primary conclusions were not dependent on Pearson distributional assumptions. GAD-7 and PHQ-9 scores were strongly positively correlated with one another (*r* = 0.824, *p* < 0.001) (Table 3).

**Table 3:** Primary participant-level association results.

Analysis	*N* occasions	Estimate	95% CI	*p* / Conclusion
Trait Index x GAD-7: Pearson r	278	−0.471	[−0.557, −0.372]	*p* < 0.001
Trait Index x PHQ-9: Pearson r	278	−0.508	[−0.595, −0.408]	p < 0.001
Trait Index x GAD-7: Spearman rho	278	−0.469	-	p < 0.001
Trait Index x PHQ-9: Spearman rho	278	−0.519	-	p < 0.001
GAD-7 x PHQ-9: Pearson r	278	0.824	-	p < 0.001
Difference: Trait-PHQ minus Trait-GAD	278	−0.037	[−0.102, 0.028]	No reliable difference
GAD-7 per +10 Trait points	278	−2.34	[−2.84, −1.83]	p < 0.001
PHQ-9 per +10 Trait points	278	−2.97	[−3.56, −2.37]	p < 0.001

Cluster-robust regression estimated that each 10-point higher Trait Index was associated with 2.34 points lower GAD-7 (95% CI 1.83 to 2.84 points lower) and 2.97 points lower PHQ-9 (95% CI 2.37 to 3.56 points lower). After adjustment for site and reported module/stage, corresponding estimates remained 2.06 points lower GAD-7 (95% CI 1.48 to 2.65 lower) and 2.81 points lower PHQ-9 (95% CI 2.14 to 3.47 lower) per 10 Trait Index points. Random-intercept mixed-effects models for participant produced closely similar estimates.

Although the Trait-PHQ-9 correlation was numerically more negative than the Trait-GAD-7 correlation by 0.037, the participant-cluster bootstrap 95% confidence interval for that difference was [−0.102, 0.028]. The data therefore did not establish a reliable difference in association strength between anxiety and depression (Figure 2).

**Figure 2:**
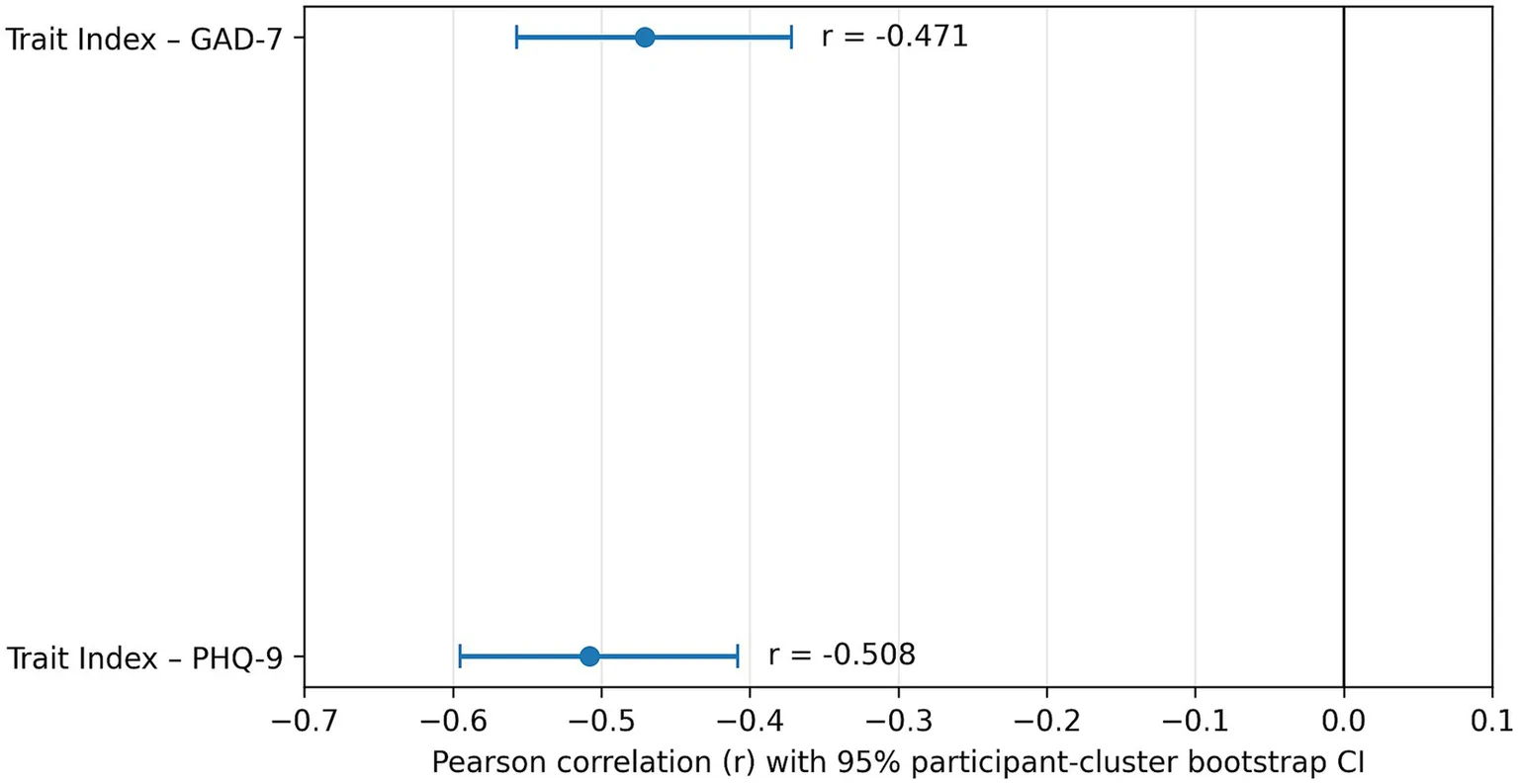
Participant-level associations between Trait Index and psychological distress. Points show Pearson correlations and horizontal bars show participant-cluster bootstrap 95% confidence intervals for 278 matched assessment occasions.

### Robustness, matching sensitivity, and linearity

Primary associations remained similar when the maximum allowable assessment span was varied from 30 min through 24 h. Trait-GAD-7 correlations ranged from −0.451 to −0.483, and Trait-PHQ-9 correlations ranged from −0.493 to −0.508 (Figure 3). Thus, the findings were not dependent on the selected 60-min primary matching window.

**Figure 3:**
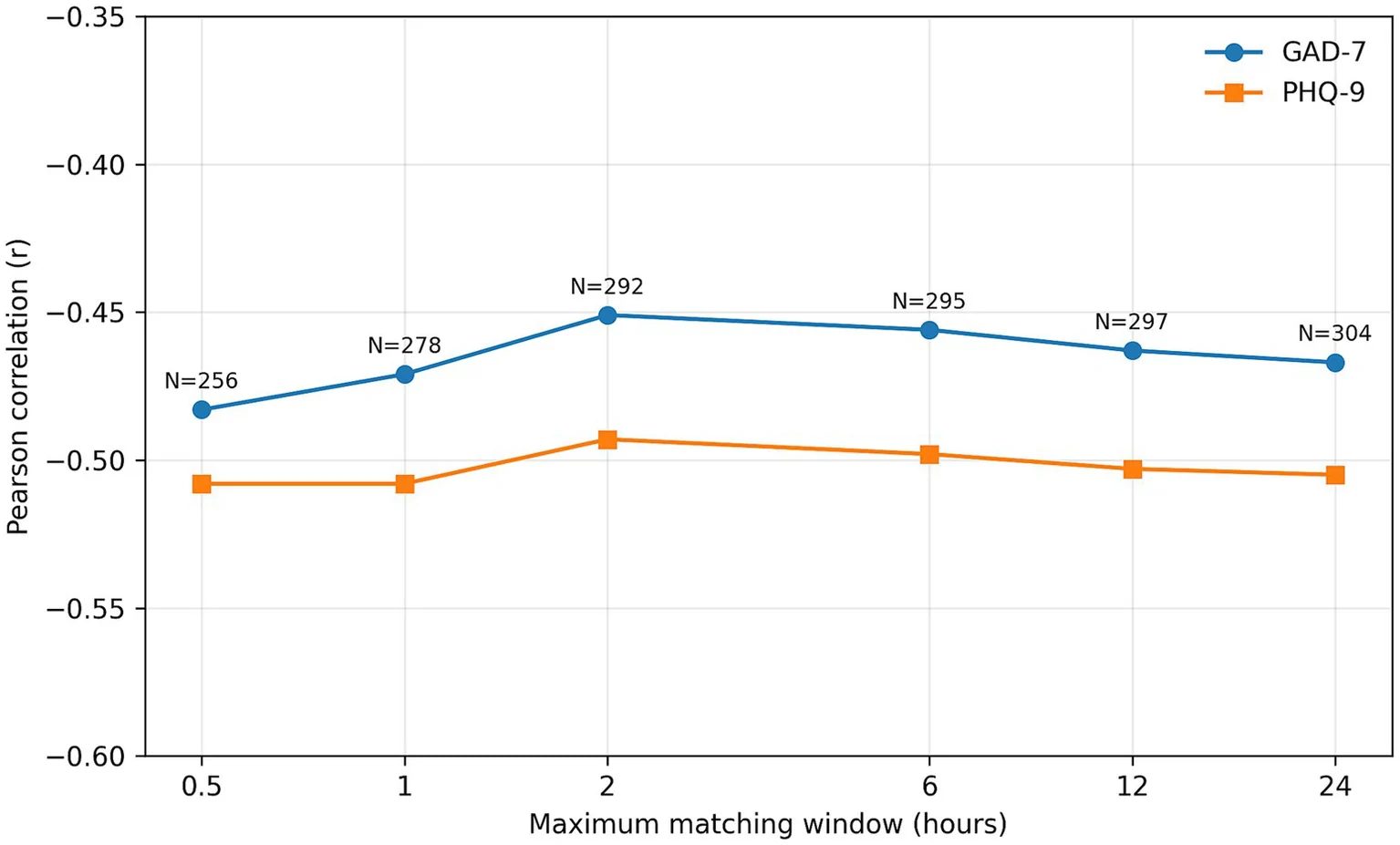
Matching-window sensitivity. Correlations remained stable when the maximum within-occasion matching span was varied from 30 min through 24 h. Labels show the number of matched occasions at each specification.

Quadratic terms did not improve fit for either outcome (GAD-7 cluster-robust quadratic *p* = 0.191; PHQ-9 *p* = 0.597). Within the observed score range, the concurrent relationships were therefore adequately represented by a linear first approximation. This finding concerns statistical form and does not imply that human development or recovery progresses linearly.

### Internal consistency of the trait assessment

The 10-domain Trait Index showed strong higher-order internal consistency across all valid Recovery administrations (alpha = 0.901; omega total = 0.901). In the identifiable current form, alpha and omega were both 0.918, and the first principal component accounted for 57.8% of domain-score variance with all 10 domains loading positively. These findings support coherence of the overall composite while also indicating that the instrument remains multidimensional.

Current-form domain reliabilities varied. Creativity (alpha = 0.840), resilience (0.797), self-awareness (0.792), tenacity (0.754), empathy (0.735), motivational orientation (0.730), and appreciation (0.719) met or approximated conventional internal-consistency expectations. Emotional intelligence (0.651), determination (0.613), and authenticity (0.565) were lower and warrant cautious subscale interpretation and further refinement. One Determination item (“I have a hard time accepting defeat”) showed minimal corrected item-total association in the current-form audit and should be evaluated in future instrument development (Table 4; Figure 4).

**Table 4:** Current-form Trait-domain internal consistency.

Trait domain	Items	*N*	Cronbach’s alpha	Bootstrap 95% CI
Tenacity	5	815	0.754	[0.725, 0.782]
Resilience	5	815	0.797	[0.771, 0.820]
Empathy	5	815	0.735	[0.692, 0.772]
Authenticity	5	815	0.565	[0.508, 0.613]
Motivational	5	815	0.730	[0.690, 0.761]
Creativity	8	812	0.840	[0.819, 0.859]
Appreciation	5	815	0.719	[0.677, 0.756]
Self-Awareness	5	815	0.792	[0.759, 0.820]
Determination	5	815	0.613	[0.563, 0.656]
Emotional Intelligence	5	815	0.651	[0.606, 0.693]

**Figure 4:**
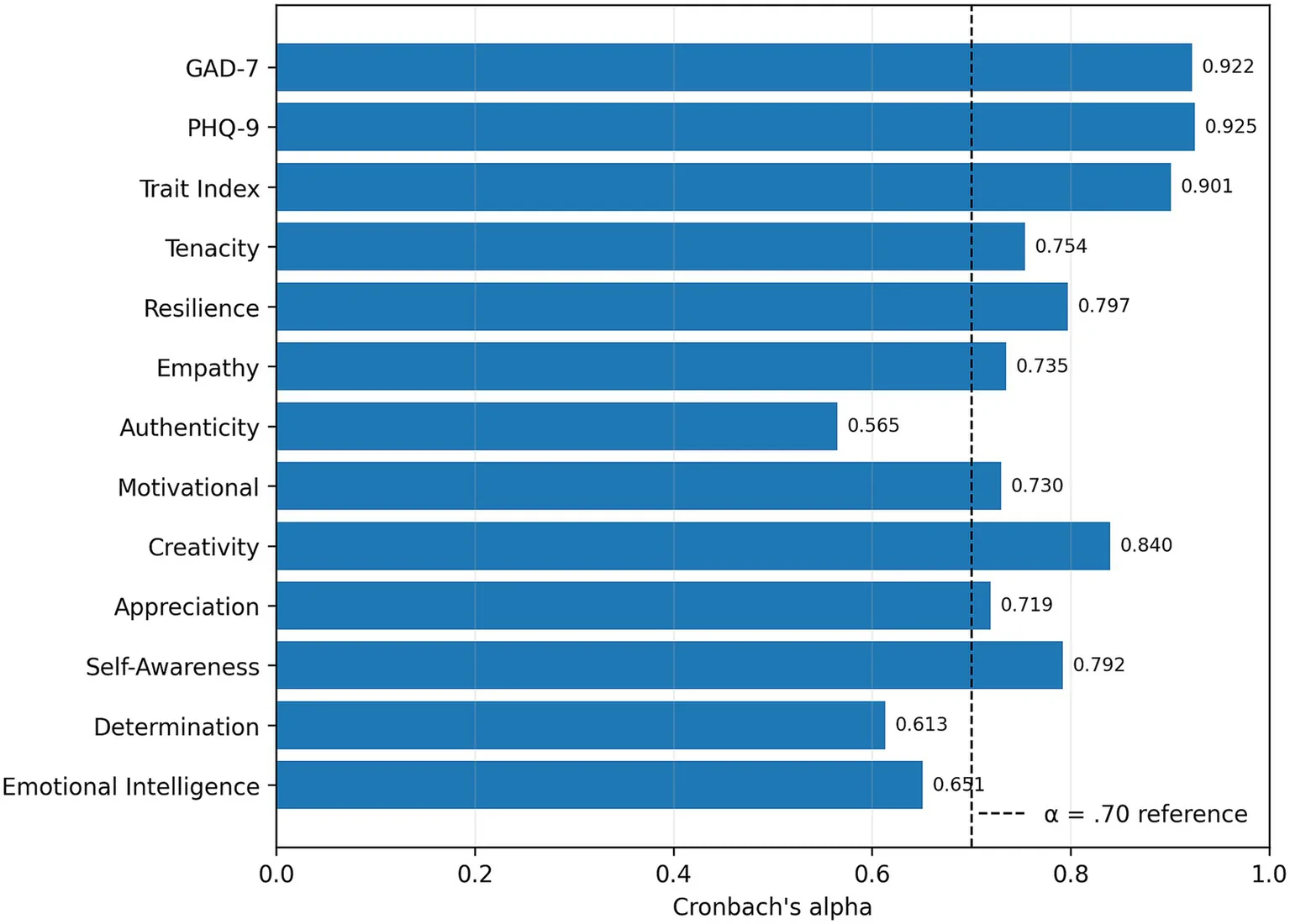
Internal consistency of primary study measures and current-form Trait domains. The dashed vertical line marks alpha = 0.70 as a descriptive reference rather than a universal validity threshold.

### Exploratory repeated-occasion analysis

Twenty-three participants had at least two non-duplicate complete matched occasions (20 with two occasions and 3 with three). The median first-to-last interval was 27.5 days (range approximately 8.0–76.5 days). From first to last matched occasion, mean Trait Index increased by 5.70 points (70.40 to 76.09; 95% CI [0.99, 10.40], *p* = 0.020). Mean GAD-7 decreased by 1.35 points (7.30 to 5.96; 95% CI [−3.00, 0.30], *p* = 0.105), and mean PHQ-9 decreased by 1.61 points (7.22 to 5.61; 95% CI [−3.85, 0.63], *p* = 0.151). Change in Trait Index was not significantly correlated with change in GAD-7 (*r* = −0.274, *p* = 0.206) or PHQ-9 (*r* = −0.081, *p* = 0.714).

Exploratory lagged regressions controlling the prior outcome value and observation interval did not identify statistically reliable temporal paths in either direction: earlier Trait Index to later GAD-7 (standardized beta = −0.091, *p* = 0.542), earlier GAD-7 to later Trait Index (beta = 0.074, *p* = 0.770), earlier Trait Index to later PHQ-9 (beta = −0.078, *p* = 0.658), or earlier PHQ-9 to later Trait Index (beta = 0.125, *p* = 0.685). Because the repeated complete sample was small, these null findings should not be interpreted as evidence that temporal relationships are absent; rather, temporal precedence remained unresolved (Table 5).

**Table 5:** Exploratory repeated-occasion and temporal-direction analyses (23 participants).

Measure / Path	Estimate / Change	95% CI	*p*
Trait Index: first to last	+5.70	[0.99, 10.40]	0.020
GAD-7: first to last	−1.35	[−3.00, 0.30]	0.105
PHQ-9: first to last	−1.61	[−3.85, 0.63]	0.151
Earlier Trait - > later GAD-7	beta = −0.091	-	0.542
Earlier GAD-7 - > later Trait	beta = 0.074	-	0.770
Earlier Trait - > later PHQ-9	beta = −0.078	-	0.658
Earlier PHQ-9 - > later Trait	beta = 0.125	-	0.685

## Discussion

The revised analysis examined the relationship between behavioral trait expression and psychological distress at the level of the same participant and assessment occasion. The principal finding was consistent across multiple analytic specifications: higher expression across the 10 Trait-Based domains was moderately associated with lower anxiety and depressive symptom severity. The participant-level associations persisted under nonparametric analysis, participant clustering, mixed-effects modeling, site/module adjustment, and alternative matching windows.

These findings align with a broader literature linking personality and trait-like characteristics to mental health while extending that question to an applied strengths-oriented behavioral framework. Prospective studies have shown that personality characteristics, especially neuroticism and related negative emotionality constructs, are associated with later anxiety, depression, and broader internalizing symptoms (Jeronimus et al., 2016; Prince et al., 2021; Conway et al., 2024). Waszczuk et al. (2022) found that personality traits contributed prognostic information about later psychiatric and functional outcomes beyond prior diagnoses, and Kang et al. (2023) demonstrated differential associations between personality traits and distinct dimensions of mental health. The present findings do not equate the Trait and Hero Discovery Assessment with traditional personality inventories, but they are consistent with the broader proposition that measurable behavioral dispositions and capacities relate meaningfully to psychological distress.

The revised data also caution against a simple symptom-specific interpretation. Trait Index associations with GAD-7 and PHQ-9 were similar in magnitude, and the direct participant-cluster comparison did not show a reliable difference between them. Anxiety and depressive scores were themselves strongly correlated. The most defensible interpretation is therefore that the overall Trait Index is related to a broader dimension of current psychological distress, while future research should determine whether particular trait domains show replicable symptom-specific patterns. Because several domain scales showed weaker internal consistency, domain-level inferences should be made more cautiously than conclusions concerning the higher-order Trait Index.

Several mechanisms could produce the observed concurrent relationships. Stronger expression of resilience, self-awareness, emotional intelligence, authenticity, determination, creativity, and related capacities could plausibly support emotion regulation, adaptive appraisal, problem solving, social connection, or persistence under stress. Conversely, anxiety and depression may temporarily constrain the accessibility or self-perceived expression of these capacities. Common underlying factors - including life stress, treatment exposure, medication, social support, site characteristics, or general response tendencies - could influence both trait and symptom scores. Common-method variance is also possible because all primary measures were self-report instruments. The present observational design cannot adjudicate among these explanations.

The exploratory repeated-occasion analysis reinforces this interpretive boundary. Trait Index scores increased significantly from first to last complete occasion in the small repeated subset, while mean GAD-7 and PHQ-9 changes were directionally lower but not statistically reliable. Lagged models did not identify a temporal path in either direction after accounting for prior values, but the repeated sample was too small for a definitive test. This is consistent with longitudinal literature suggesting that trait-distress relationships can reflect vulnerability, state effects, and reciprocal processes rather than a single fixed sequence (Prince et al., 2021; Kang et al., 2023). Establishing antecedence requires prospectively scheduled repeated measurements and substantially larger within-person samples.

The findings have practical implications for behavioral healthcare assessment, but they should be interpreted conservatively. The Trait Index is not a diagnostic instrument and should not replace established symptom screening such as the GAD-7 or PHQ-9. Instead, a strengths-oriented trait measure may provide complementary information about capacities that participants and clinicians can discuss alongside symptoms. In applied recovery settings, such information may help broaden assessment beyond deficit and symptom language while preserving validated clinical screening. Whether measuring or deliberately strengthening these capacities improves mental-health outcomes remains an empirical question for prospective and controlled research.

### Limitations

Several limitations should be considered when interpreting these findings. First, the study used retrospective observational data collected during routine behavioral health programming. Although the revised participant-level analysis demonstrates consistent associations between trait expression and anxiety and depressive symptoms, the design does not permit causal inference. Higher trait expression may contribute to lower psychological distress, psychological distress may influence the expression or accessibility of behavioral traits, or both may be influenced by unmeasured contextual factors. The present findings therefore establish association rather than temporal or causal direction.

Second, although the primary analysis matched assessments to the same participant and assessment occasion and accounted for repeated observations, only a relatively small subset of participants had multiple complete matched occasions across the Trait Assessment, GAD-7, and PHQ-9. Exploratory longitudinal analyses among this subset did not establish whether changes in trait expression preceded later changes in anxiety or depression, or whether changes in psychological distress preceded later trait expression. These analyses were limited by sample size and variable intervals between assessments and should not be interpreted as definitive tests of temporal precedence.

Third, the assessments were administered under naturalistic program conditions rather than a standardized research schedule. Participants progressed through programming at different rates, and assessment timing varied across individuals and sites. Although this variability reflects real-world behavioral healthcare implementation and enhances ecological relevance, it limits control over treatment exposure, assessment interval, and other contextual influences. Factors such as life stress, medication, concurrent treatment, social support, treatment intensity, and site-specific practices were not consistently available and may partially account for the observed relationships.

Fourth, all primary measures were self-report instruments. The GAD-7 and PHQ-9 demonstrated strong internal consistency in the present sample, and the overall Trait Index also demonstrated strong internal consistency. However, several individual Trait Assessment domains showed comparatively lower reliability and should be interpreted cautiously. Further psychometric evaluation of the Trait and Hero Discovery Assessment, including factor structure, measurement invariance, test–retest reliability, and sensitivity to developmental change, is warranted.

Fifth, participant records were linked retrospectively using available identifying information before creation of the coded analytic dataset. Although conservative matching and quality-control procedures were used and findings remained stable across alternative matching windows, some possibility of record-linkage error remains. Additionally, requiring complete matched data across the three instruments may introduce selection bias if participants who completed all assessments differed systematically from those who did not.

Finally, the sample consisted of individuals participating in recovery-oriented behavioral healthcare programming across multiple U. S. settings. The multisite nature of the data increases real-world relevance, but findings may not generalize to populations outside recovery services, to individuals not engaged in structured behavioral health programming, or to other age and demographic groups. Replication in independent clinical, prevention, and community samples will be necessary to determine the broader generalizability of the observed associations.

### Future research

The present findings support several directions for continued investigation. Most importantly, future studies should examine the temporal relationship between trait expression and psychological distress using prospective longitudinal designs with standardized assessment intervals. The current study demonstrates that higher trait expression is associated with lower anxiety and depressive symptom severity at matched assessment occasions, but it cannot determine whether changes in trait expression precede changes in psychological distress, whether psychological distress alters the expression of behavioral traits, or whether the relationship is reciprocal.

A particularly important next step will be to follow participants across repeated baseline, mid-program, post-program, and longer-term follow-up assessments. Such designs would permit examination of within-person trajectories and allow lagged or cross-lagged analyses testing whether earlier trait expression predicts subsequent anxiety or depression after accounting for prior symptom severity, and whether earlier psychological distress similarly predicts subsequent trait expression. This would move the research from identifying concurrent association toward evaluating possible developmental sequence and mechanism.

Future work should also examine whether individual trait domains contribute differently to psychological functioning over time. The present study focused primarily on the overall Trait Index because it demonstrated stronger psychometric consistency than several individual domains. Larger prospective samples could clarify whether particular capacities - such as resilience, self-awareness, emotional intelligence, authenticity, determination, or creativity - function as distinct protective factors, developmental mechanisms, or indicators of broader psychological adaptation.

Further psychometric research on the Trait and Hero Discovery Assessment is also warranted. This should include confirmatory and exploratory factor analyses, test–retest reliability, measurement invariance across populations and settings, sensitivity to change, and continued refinement of individual domain scales with lower internal consistency. Independent validation samples will be particularly important given that the instrument was developed within the Trait-Based framework examined in the present study.

Finally, replication across populations outside recovery-oriented behavioral healthcare will help determine the generalizability of the observed relationship between trait expression and psychological distress. Separate studies involving prevention populations, community samples, educational settings, and other behavioral health contexts could examine whether similar patterns emerge across developmental stages and levels of clinical severity. Collectively, such research may clarify whether behavioral trait expression represents a relatively broad dimension of psychological functioning and whether changes in these capacities have prospective relevance for mental health, adaptation, and behavioral outcomes.

## Conclusion

The present study provides evidence that behavioral trait expression is meaningfully associated with psychological distress at the individual level within recovery-oriented behavioral healthcare settings. Across matched assessment occasions, higher overall Trait Index scores were consistently associated with lower anxiety and depressive symptom severity, and these relationships remained stable across multiple analytic approaches and sensitivity tests.

These findings support a perspective in which psychological functioning may be understood not only through the presence or severity of symptoms, but also through the broader behavioral capacities available to individuals as they respond to stress, adversity, and change. Traits such as resilience, self-awareness, emotional intelligence, authenticity, determination, and creativity may therefore represent important dimensions of functioning that complement traditional symptom-focused assessment.

At the same time, the observational design does not establish whether stronger trait expression reduces psychological distress, whether distress constrains the expression of these capacities, or whether the relationship operates reciprocally. Determining this temporal direction represents an important next step for longitudinal research.

Taken together, the findings support continued investigation of behavioral trait expression as a potentially meaningful component of psychological functioning. Integrating attention to strengths and capacities alongside symptom assessment may offer a more complete understanding of individuals within applied behavioral health settings and provide a foundation for future research examining the developmental mechanisms through which psychological adaptation and distress interact over time.

## Data Availability

The data analyzed in this study is subject to the following licenses/restrictions: the dataset contains de-identified participant data collected within recovery-oriented behavioral healthcare settings. Due to privacy, ethical, and organizational considerations, the data are not publicly available. Aggregated data may be made available by the corresponding author upon reasonable request. Requests to access these datasets should be directed to JR, Jason@StartfromStrength.org.

## References

[ref1] Borsboom D. (2017). A network theory of mental disorders. World Psychiatry 16, 5–13. doi: 10.1002/wps.20375, 28127906 PMC5269502

[ref2] Conway C. C. Grogans S. E. Anderson A. S. Islam S. Craig L. E. Wedlock J. . (2024). Neuroticism is prospectively associated with 30-month changes in broadband internalizing symptoms, but not narrowband positive affect or anxious arousal, in emerging adulthood. Clin. Psychol. Sci. 12, 823–839. doi: 10.1177/21677026231205270, 39359716 PMC11446481

[ref3] GBD 2019 Mental Disorders Collaborators (2022). Global, regional, and national burden of 12 mental disorders in 204 countries and territories, 1990-2019: a systematic analysis for the global burden of disease study 2019. Lancet Psychiatry 9, 137–150. doi: 10.1016/S2215-0366(21)00395-3, 35026139 PMC8776563

[ref4] Hayes S. C. Hofmann S. G. Ciarrochi J. (2020). A process-based approach to psychological diagnosis and treatment: the conceptual and treatment utility of an extended evolutionary meta model. Clin. Psychol. Rev. 82:101908. doi: 10.1016/j.cpr.2020.101908, 32932093 PMC7680437

[ref5] Jeronimus B. F. Kotov R. Riese H. Ormel J. (2016). Neuroticism's prospective association with mental disorders halves after adjustment for baseline symptoms and psychiatric history, but the adjusted association hardly decays with time: a meta-analysis on 59 longitudinal/prospective studies with 443,313 participants. Psychol. Med. 46, 2883–2906. doi: 10.1017/S0033291716001653, 27523506

[ref6] Kang W. Steffens F. Pineda S. Widuch K. Malvaso A. (2023). Personality traits and dimensions of mental health. Sci. Rep. 13:7091. doi: 10.1038/s41598-023-33996-1, 37127723 PMC10151354

[ref7] Kroenke K. Spitzer R. L. Williams J. B. W. (2001). The PHQ-9: validity of a brief depression severity measure. J. Gen. Intern. Med. 16, 606–613. doi: 10.1046/j.1525-1497.2001.016009606.x, 11556941 PMC1495268

[ref8] McCrae R. R. Costa P. T. Jr. (2008). “The five-factor theory of personality,” in Handbook of Personality: Theory and Research, eds. John O. P. Robins R. W. Pervin L. A.. 3rd ed (New York, NY: Guilford Press), 159–181.

[ref9] Prince E. J. Siegel D. J. Carroll C. P. Sher K. J. Bienvenu O. J. (2021). A longitudinal study of personality traits, anxiety, and depressive disorders in young adults. Anxiety Stress Coping 34, 299–307. doi: 10.1080/10615806.2020.1845431, 33190525 PMC8068574

[ref10] Roberts B. W. Walton K. E. Viechtbauer W. (2006). Patterns of mean-level change in personality traits across the life course: a meta-analysis of longitudinal studies. Psychol. Bull. 132, 1–25. doi: 10.1037/0033-2909.132.1.1, 16435954

[ref11] Spitzer R. L. Kroenke K. Williams J. B. W. Löwe B. (2006). A brief measure for assessing generalized anxiety disorder: the GAD-7. Arch. Intern. Med. 166, 1092–1097. doi: 10.1001/archinte.166.10.1092, 16717171

[ref12] Stieger M. Flückiger C. Rüegger D. Kowatsch T. Roberts B. W. Allemand M. (2021). Changing personality traits with the help of a digital personality change intervention. Proc. Natl. Acad. Sci. USA 118:e2017548118. doi: 10.1073/pnas.2017548118, 33558417 PMC7923371

[ref13] Waszczuk M. A. Hopwood C. J. Luft B. J. Morey L. C. Perlman G. Ruggero C. J. . (2022). The prognostic utility of personality traits versus past psychiatric diagnoses: predicting future mental health and functioning. Clin. Psychol. Sci. 10, 734–751. doi: 10.1177/21677026211056596, 35967764 PMC9366938

